# Analysis of diversity and function of epiphytic bacterial communities associated with macrophytes using a metagenomic approach

**DOI:** 10.1007/s00248-024-02346-7

**Published:** 2024-01-29

**Authors:** Xin Wang, Yi Liu, Chun Qing, Jin Zeng, Jixing Dong, Pinhua Xia

**Affiliations:** 1 Guizhou Province Key Laboratory for Information System of Mountainous Areas and Protection of Ecological Environment, Guizhou Normal University, Guiyang, 550025 China; 2 Guizhou Caohai National Nature Reserve Management Committee, Weining, 55310 China; 3 State Key Laboratory of Lake Science and Environment, Nanjing Institutie of Geography and Limnology, Chinese Academy of Science, Nanjing, 210008 China

**Keywords:** Submerged macrophytes, Epiphytic bacteria, Lake ecosystem, Diversity, Functional traits

## Abstract

Epiphytic bacteria constitute a vital component of aquatic ecosystems, pivotal in regulating elemental cycling. Despite their significance, the diversity and functions of epiphytic bacterial communities adhering to various submerged macrophytes remain largely unexplored. In this study, we employed a metagenomic approach to investigate the diversity and function of epiphytic bacterial communities associated with six submerged macrophytes: *Ceratophyllum demersum*, *Hydrilla verticillata*, *Myriophyllum verticillatum*, *Potamogeton lucens*, *Stuckenia pectinata*, and *Najas marina*. The results revealed that the predominant epiphytic bacterial species for each plant type included *Pseudomonas* spp., *Microbacterium* spp., and *Stenotrophomonas rhizophila*. Multiple comparisons and linear discriminant analysis effect size indicated a significant divergence in the community composition of epiphytic bacteria among the six submerged macrophytes, with 0.3–1% of species uniquely identified. Epiphytic bacterial richness associated with *S. pectinata* significantly differed from that of both *C. demersum* and *H. verticillata*, although no significant differences were observed in diversity and evenness. Functionally, notable variations were observed in the relative abundances of genes associated with carbon, nitrogen, and phosphorus cycling within epiphytic bacterial communities on the submerged macrophyte hosts. Among these communities, *H. verticillata* exhibited enrichment in genes related to the 3-hydroxypropionate bicycle and nitrogen assimilation, translocation, and denitrification. Conversely, *M. verticillatum* showcased enrichment in genes linked to the reductive citric acid cycle (Arnon-Buchanan cycle), reductive pentose phosphate cycle (Calvin cycle), polyphosphate degradation, and organic nitrogen metabolism. In summary, our findings offer valuable insights into the diversity and function of epiphytic bacteria on submerged macrophyte leaves, shedding light on their roles in lake ecosystems.

**Supplementary Information:**

The online version contains supplementary material available at 10.1007/s00248-024-02346-7.

## Introduction

Submerged macrophytes, as major primary producers in lake ecosystems, play a crucial role in shaping the structure and functioning of shallow lake ecosystems. The leaves of these submerged macrophytes offer diverse ecological niches for microorganisms [1]. Epiphytic bacterial communities associated with these macrophytes influence plant physiology and nutrient cycling in lake ecosystems, encompassing processes such as carbon, nitrogen, and phosphorus uptake and transformation, as well as heavy metal adsorption [2–4]. Thus, understanding the composition and function of epiphytic bacterial communities on submerged plant leaves is crucial for comprehending nutrient cycling in lakes.

The structure of epiphytic bacterial communities adhering to seaweed and terrestrial plants typically displays host-specific characteristics demonstrating a strong filtering effect of host plants on epiphytic bacteria [5, 6], revealing that foliar-attached bacterial communities in arid deserts primarily consist of *Actinobacteria*, *Firmicutes*, and *Proteobacteria*. Liu et al. reported significant diversity differences in leaf surface epiphytic bacterial communities among nine woodland tree species, displaying varying functional traits related to plant species [6]. *Proteobacteria*, *Bacteroidota*, *Verrucomicrobia*, *Planctomycetota*, *Firmicutes*, *Patescibacteria*, and *Cyanobacteria* constitute the core of epiphytic bacterial communities attached to marine macroalgae [7]. However, epiphytic bacterial communities on different algae surfaces exhibit host specificity [5, 8]. Despite extensive studies on terrestrial plants and marine algae [6, 9–12], the exploration of aquatic epiphyton gained momentum only in the early twentieth century. Consequently, there is a substantial knowledge gap regarding the composition and variability of epiphytic bacterial communities associated with different submerged macrophytes in freshwater lakes. Goldsborough et al. proposed that leaf surfaces of submerged macrophytes are not neutral substrates for epiphytic bacteria [13]. The establishment of a plant-bacterial interface depends on the physicochemical properties of plant leaves and the ability of bacteria to adapt to and modify this environment. Thus, morphological or metabolic differences in plant leaf surfaces can result in host-specific epiphytic bacterial communities [14]. For example, it was suggested that plant foliar sugar content limits the epiphytic bacterial population size under favorable environmental conditions [15]. Hempel et al. examined bacterial community composition on leaves of *Chara aspera* and *Myriophyllum spicatum* using FISH, revealing variability in colonizing bacterial communities [16]. He et al. found significant differences in epiphytic bacterial communities in the leaves of the high-phenolic plant *Hydrilla verticillata* and the low-phenolic plant *Vallisneria natans* using terminal restriction fragment length polymorphism (T-RFLP) and clone library analysis targeting bacterial 16S rRNA genes [17]. Differences in phenolic content among plant species may be a major factor contributing to the differences in epiphytic bacterial communities [3]. In contrast, epiphytic bacterial communities of *M. spicatum* from different locations exhibited similar core bacterial species [18], suggesting that conspecifics maintain similar core epiphytic bacterial communities despite environmental variations. However, our knowledge on this topic remains limited.

Epiphytic bacteria play a crucial role in the metabolic cycle of freshwater ecosystems, demonstrating diverse mechanisms of nitrogen metabolism, including nitrogen fixation, nitrification, and denitrification in bacterial communities on plant stems and leaves [19]. Yan et al. confirmed the excellent denitrification capacity of three submerged epiphytic bacterial communities [20], and Sun et al. identified functions related to nitrogen metabolism in *M. spicatum* epiphytic bacteria. While these studies deepen our understanding of the biogeochemical cycle driven by epiphytic bacteria on submerged macrophytes in lake ecosystems, most previous research has focused on comparisons between only two or three submerged macrophytes [18]. For example, He et al. analyzed differences in the epiphytic bacterial communities of two submerged macrophytes, *H. verticillata* and *V. natans* [17]. With the advancement of macrogenome sequencing technology, high-throughput sequencing allows for a more comprehensive exploration of epiphytic bacterial community diversity on submerged macrophytes and the determination of metabolic pathways of environmental microorganisms [21]. Nevertheless, the functions of epiphytic bacterial communities on submerged macrophytes remain largely unknown.

This study aims to explore the compositional and functional variability of epiphytic bacterial communities on the leaf surfaces of six different species of submerged macrophytes using macrogenomics. Our objectives include understanding the (1) structure and diversity of community composition of epiphytic bacteria in different plants and their correlation with plant complexity, (2) differences in the structure of host plant-attached microbial communities, and (3) differences in metabolic genes associated with nutrient cycling in epiphytic bacterial communities among different host plants. Investigating the structure and function of epiphytic bacterial communities on submerged plant communities not only enriches the understanding of their biodiversity but also provides insights into the role of submerged macrophytes in lake ecological restoration.

## Materials and Methods

### Study Area and Sampling

Weining Caohai (26° 47′ 35″ N, 104° 9′ 23″ E) in Guizhou Province is a complete and representative karst plateau wetland ecosystem, formed by water accumulation in karst basins. It stands as one of the few natural freshwater lakes at the same latitude on the plateau. Located in the hinterland of the Wumeng Mountains on the Yunnan-Guizhou Plateau, on the northwest edge of Guizhou Province, it spans 25 km^2^ with an elevation of 2171.7 m. Known as the “underwater forest”, Caohai Lake is abundant in submerged vegetation. In October 2021, we gathered 36 biofilm samples from the leaves of six submerged plant species at a 0.5 m water depth from six sites in the southwestern part of Weining Caohai, where aquatic plants flourish. Each site harbored six species of submerged plants, including *Ceratophyllum demersum*, *Hydrilla verticillata*, *Myriophyllum verticillatum*, *Potamogeton lucens*, *Stuckenia pectinata*, and *Najas marina*. Sampling sites, spaced 1 km apart with similar hydrogeological conditions, hosted 3–5 replicate plants of each species. Approximately 10 g of leaves were cut and transferred to 500 ml sterile polyethylene bottles containing 400 ml of 0.05 M phosphate-buffered saline (PBS, pH = 7.4) for epiphytic bacteria analysis [22]. All leaf samples were collected in triplicate, mixed, stored on ice packs, and promptly returned to the laboratory. The samples were sonicated for 3 min, shaken for 30 min, and then sonicated again for 3 min before being retrieved with forceps. An aliquot of the remaining eluate (100 ml) was then filtered through a 0.22 μm membrane to obtain epiphytic bacteria samples.

### DNA Extraction and PCR Amplification and Sequencing

DNA extraction from the collected leaf epiphytic bacteria samples utilized the E.Z.N.A. ® Soil DNA Kit (Omega Biotek, Norcross, GA, USA). DNA concentration was measured using a Quantus Fluorometer (Picogreen), DNA purity using a NanoDrop2000, and DNA integrity using 1% agarose gel electrophoresis. Extracted DNA was fragmented using a Covaris M220 Focused-ultrasonicator (Genomics, China) and screened for fragments of approximately 400 bp. Paired-end libraries were constructed using NEXTFLEX Rapid DNA-Seq (Bio Scientific, Austin, TX, USA) and sequenced using NovaSeq Reagent Kits at Majorbio Bio-Pharm Technology Co., Ltd. (Shanghai, China). Macrogenome sequencing was performed using an Illumina NovaSeq 6000 (Illumina, USA) platform.

### Sequence Data Processing

Raw data underwent quality control using Fastp software; reads were aligned to host plant DNA sequences with Burrows-Wheeler Alignment (BWA) software, and highly similar contaminated reads were eliminated. Optimized sequences were spliced and assembled with MEGAHIT based on succinct de Bruijn graphs, filtering contigs ≥ 400 bp as the final assembly results. Open reading frame (ORF) prediction for assembled contigs utilized Prodigal v2.6.3. Predicted gene sequences were clustered with CD-HIT software to construct a non-redundant gene set. SOAPaligner software compared high-quality reads of each sample with the non-redundant gene set (95% identity), and gene abundance was counted. Species annotation results were obtained from the corresponding taxonomic information database of NR (non-redundant protein sequence database). Gene abundance at each taxonomic level, i.e., domain, kingdom, phylum, class, order, family, genus, and species, was calculated. A *P* < 0.05 was considered statistically significant.

### Statistical Analyses

Differences in gene abundance and α-diversity of epiphytic bacteria between groups were analyzed using the Kruskal-Wallis rank-sum tests, followed by the Tukey-Kramer post hoc tests. Dominant species and functional differences in epiphytic bacteria of different plants were determined using the Kruskal-Wallis test (nonparametric approach) [23]. Venn diagrams were generated to analyze the shared and endemic epiphytic bacteria among the six submerged macrophytes. Principal coordinate analysis (PCoA) and non-metric multidimensional scaling analysis (NMDS) based on the Bray-Curtis distance were performed using the “vegan” package. Beta diversity matrix calculations were performed to evaluate differences in the detection of epiphytic bacteria at the species level [24]. Linear discriminant analysis effect size (LEfSe) identified epiphytic bacterial species differing between groups, with LDA > 3.5 as the threshold value [25]. To explore bacterial community metabolism, BLASTP was used with the Kyoto Encyclopedia of Genes and Genomes (KEGG) database to obtain KEGG annotation profiles corresponding to genes and potential metabolic pathways. The total number of identified protein features (i.e., predicted protein-coding regions) was used to normalize the relative abundance of metabolism-related genes and to generate heatmap plots to compare the abundance of genes in different epiphytic bacteria. All analyses were performed using R 4.1.2.

## Results

### Community Structure and Diversity of Epiphytic Bacteria

**Fig. 1:**
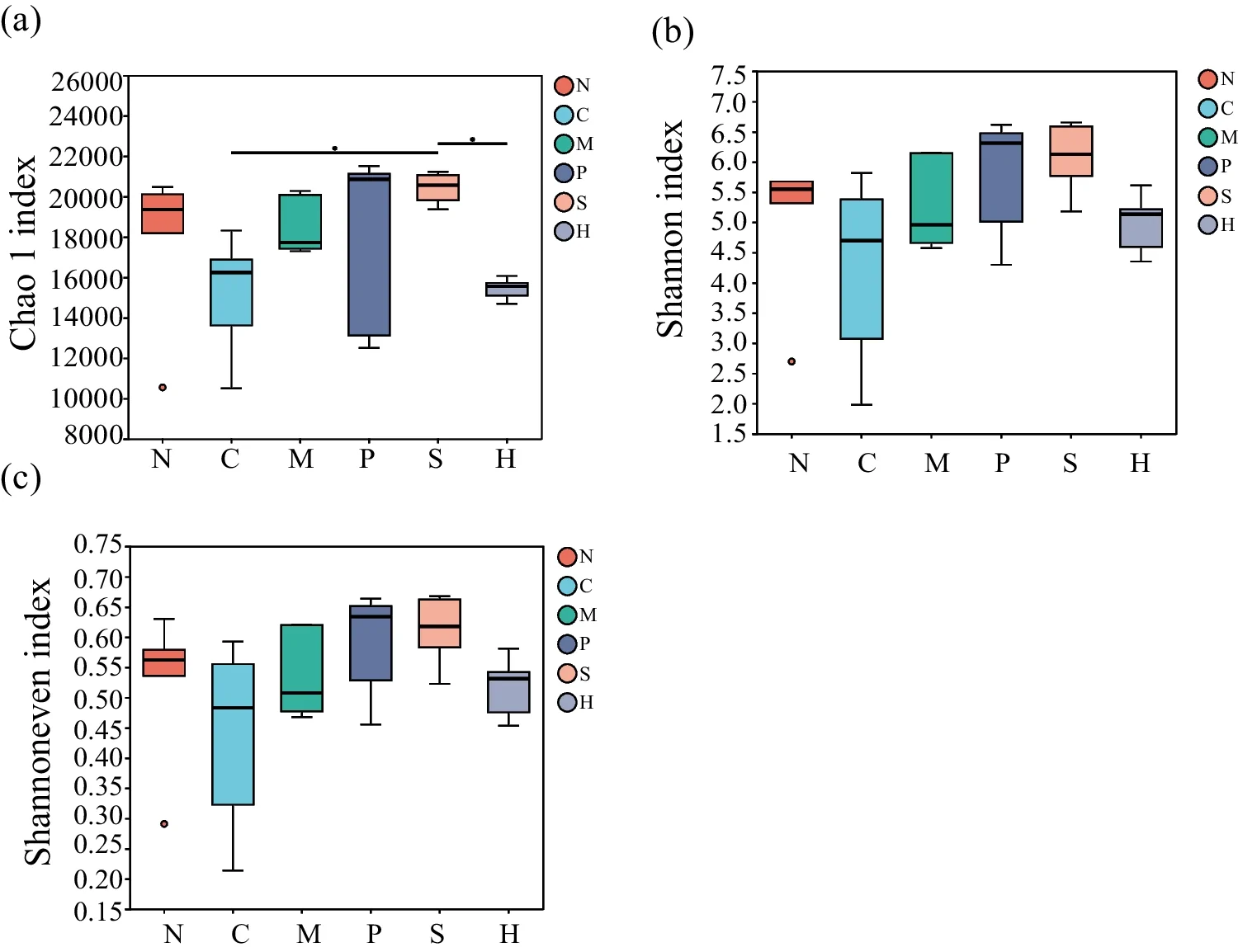
Alpha diversity of plant epiphytic bacterial samples estimated by **a** Chao index, **b** Shannon index, and **c** Shannon even index. Each box indicates the 3rd and 1st quartiles of the top and bottom boundary value ranges, respectively, and the black line within each box indicates the median value. Statistical comparison of indicated data was performed using the Kruskal-Wallis rank-sum test with *(*P* < 0.05). N, *N. marina*; P, *P. lucens*; M, *M. verticillatum*; C, *C. demersum*; H, *H. verticillata*; S, *S. pectinata*; there were 6 replicates for each plant

Metagenomic data comprised 1,819,941,160 raw reads, with 1,767,793,646 clean reads generated through the trimming and filtering of lower-quality data. After quality control, the sequence length of clean reads accounted for approximately 97% of the original sequence length, indicating high overall data quality. MEGAHIT splicing yielded 127,876,660 assembled sequences (contigs), with an average total sequence length of 261 Mb per sample. The coverage was close to 1, and the Rarefaction curve of the species exhibited a plateau, indicating sufficient sample size and data depth. Epiphytic bacterial richness (Chao1) attached to *S. pectinata* significantly surpassed that of *C. demersum* and *H. verticillata* (*P* < 0.05). The Shannon and Shannon even indices showed that the diversity and evenness of the *S. pectinata* epiphytic bacterial community were more diverse and homogeneous than those of *C. demersum*, though not significantly different (*P* > 0.05) (Fig. 1).

**Fig. 2:**
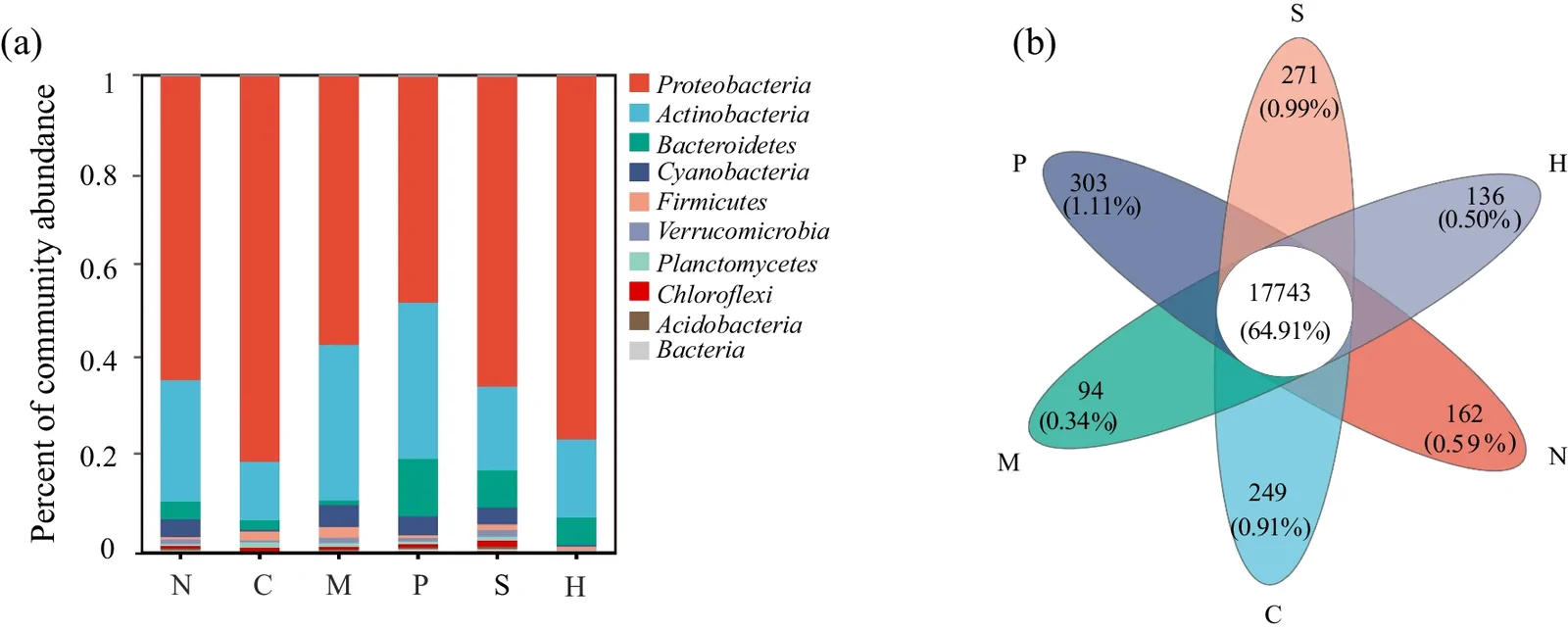
**a** Relative abundance of epiphytic bacteria at species level of six submerged macrophytes. **b** Number of shared and unique taxa at the species level of six plant epiphytic bacteria. N, *N. marina*; P, *P. lucens*; M, *M. verticillatum*; C, *C. demersum*; H, *H. verticillata*; S, *S. pectinata*

Based on comparisons with the NR database, 149 bacterial phyla were detected, encompassing 240 classes, 413 orders, 814 families, 3312 genera, and 27,336 species. In 36 samples, *Proteobacteria* was the prevailing phylum across all plants. *Pseudomonadaceae* was associated with each plant, at varying percentages: *C. demersum* (47.05%), *H. verticillata* (26.45%), and *N. marina* (15.45%) (Fig. 2a). The dominant family differed for *M. verticillatum*, *P. lucens*, and *S. pectinata*, being *Microbacteriaceae* (16.75%), *Micrococcaceae* (11.45%), and *Xanthomonadaceae* (10.23%), respectively. While the composition of epiphytic bacterial communities was similar, the abundance varied among plants at the genus level. For example, the abundance of *Pseudomonas* was the highest in *C. demersum*, *H. verticillata*, and *N. marina* (56.26%, 29.10%, and 20.26%, respectively), while *Microbacterium* was the most abundant in *M. verticillatum* and *P. lucens* (17.01% and 11.71%, respectively), and *Stenotrophomonas* dominated in *S. pectinata* (12.79%). At the species taxonomic level, *C. demersum* and *H. verticillata* were dominated by *Pseudomonas* spp. (26.63% and 14.67%, respectively); *M. verticillatum* by species of *Pseudomonas* spp. (7.27%), *Microbacterium* (5.47%), and *Sphingomonas* (4.45%); *P. lucens* by *Microbacterium* (2.25%); *S. pectinata* by *Stenotrophomonas_rhizophila* (6.97%); and *N. marina* by *Pseudomonas* spp. (8.90%) and *Massilia* sp. (5.66%).

The habitat heterogeneity hypothesis holds that habitat complexity may positively affect the abundance of attached organisms, while the microhabitat hypothesis holds that plant complexity has an important impact on the abundance of attached organisms, community diversity, and species composition changes [26, 27]. Fractal dimension was introduced to characterize plant complexity [28, 29]. Correlation analysis was used to explore the correlation between the diversity of epiphytic bacterial communities and the leaf shape dimension of six submerged plants (Fig. S1). The fractal dimension was only significantly correlated with the Shannon index (*P* < 0.05).

### Differences in Epiphytic Bacterial Communities of Different Submerged Macrophytes

**Fig. 3:**
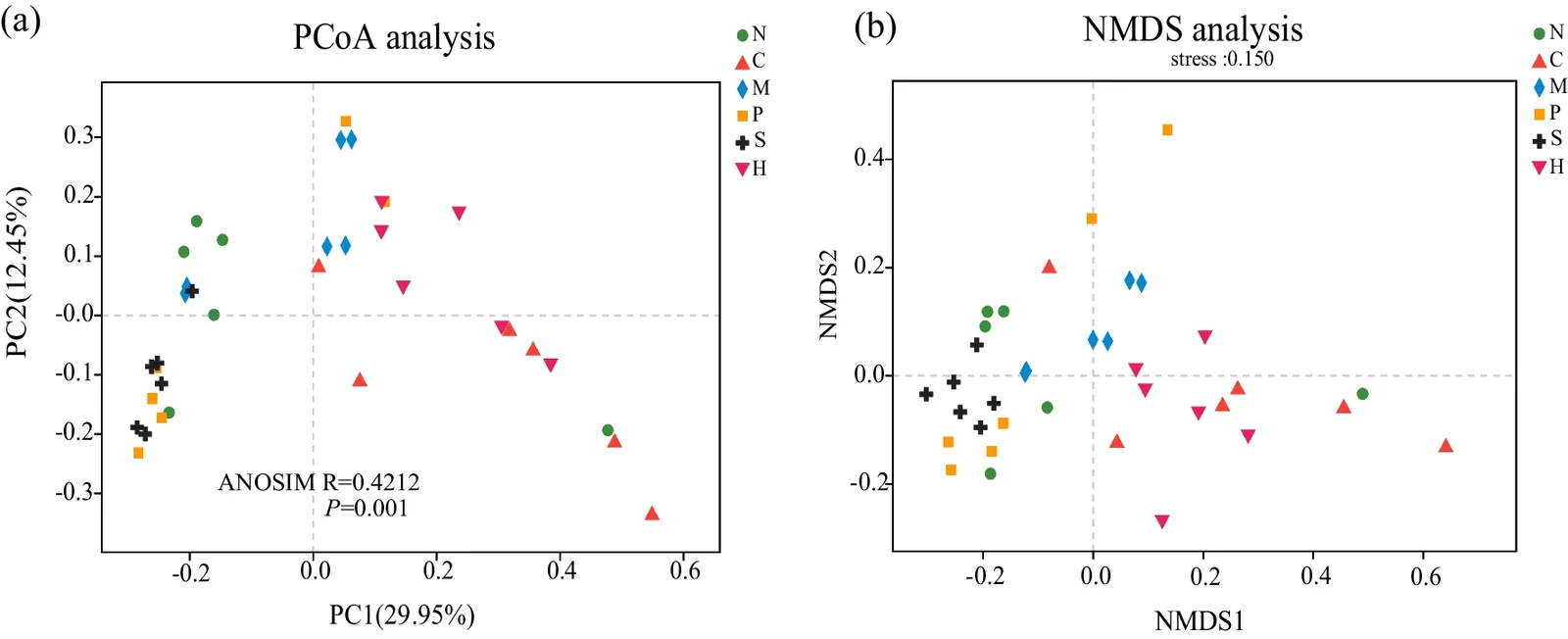
**a** Principal component analysis (PCoA) and **b** non-metric multidimensional scaling (NMDS) analysis showing differences in the composition of the epiphytic bacterial communities of the six submerged macrophytes at the species level (for Bray-Curtis). N, *N. marina*; P, *P. lucens*; M, *M. verticillatum*; C, *C. demersum*; H, *H. verticillata*; S, *S. pectinata*

A Venn diagram analyzing epiphytic bacterial communities at the family to genus taxonomic levels revealed one unique family present in *C. demersum* (*Candidatus_Actinomarinaceae*), *P. lucens* (*Candidatus_Niyogibacteria*), and *S. pectinata* (*Defluviitaleaceae*) (Fig. S2a and b). At the genus level, *P. lucens* displayed the highest number (22) of unique bacteria, with *Mesoplasma* accounting for the largest proportion (14.67%). At the species level, the number of species endemic to each plant was as follows: *C. demersum* (249), *H. verticillata* (136), *M. verticillatum* (94), *P. lucens* (303), *S. pectinata* (271), and *N. marina* (162) (Fig. 2b). PCoA and analysis of similarities (ANOSIM) indicated statistically significant differences in the composition of epiphytic bacterial communities among plant species (*R* = 0.4212, *P* = 0.001) (Fig. 3a). This result was confirmed using NMDS (stress = 0.150) (Fig. 3b).

**Fig. 4:**
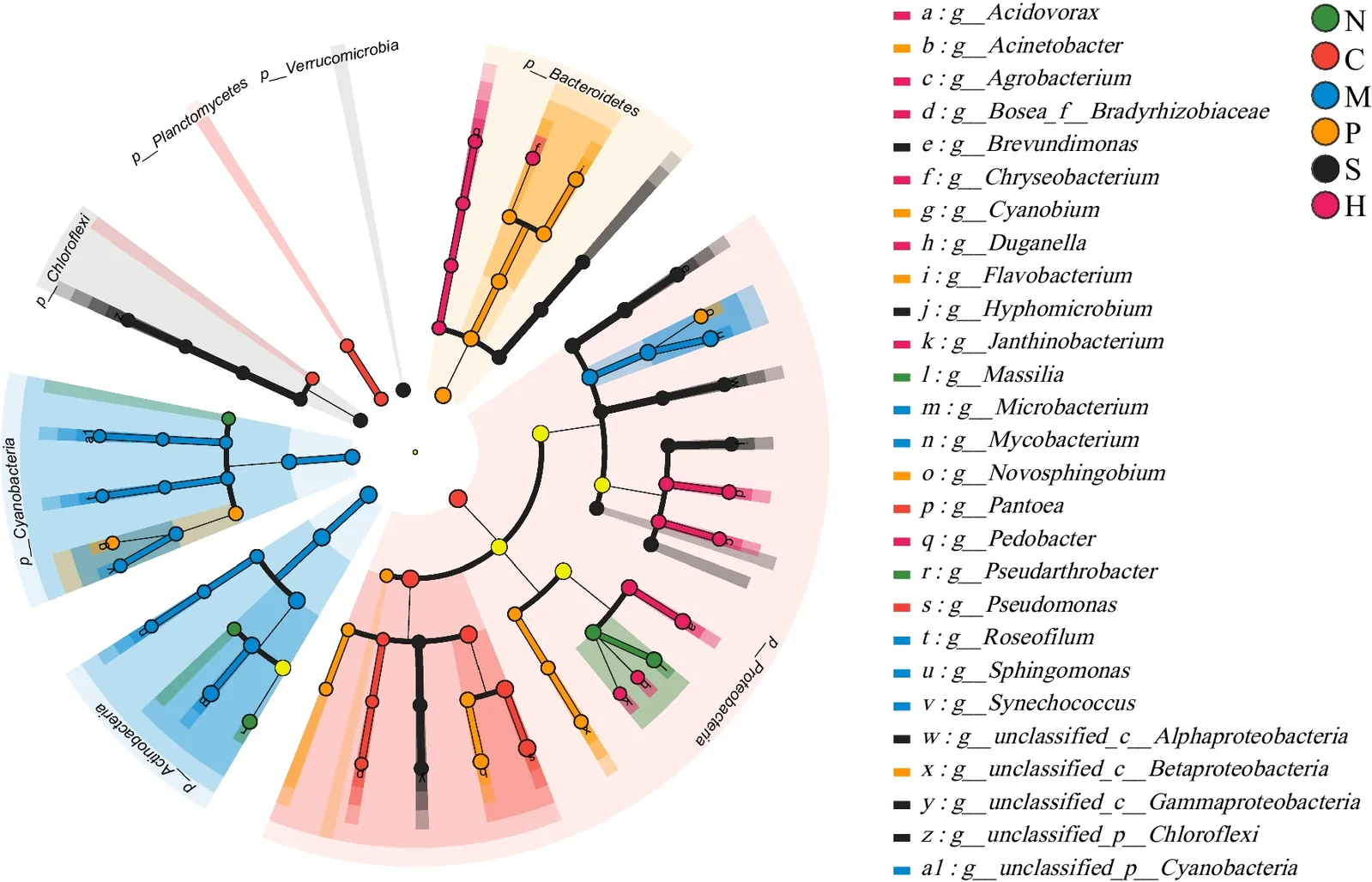
Different colored nodes in the LEfSe plot indicate microbial taxa that are significantly enriched in the corresponding group and have a significant effect on intergroup differences; light yellow nodes indicate microbial taxa that are not significantly different in any of the different subgroups or have no significant effect on intergroup differences. N, *N. marina*; P, *P. lucens*; M, *M. verticillatum*; C, *C. demersum*; H, *H. verticillata*; S, *S. pectinata*

LEfSe revealed detailed differences in the distribution of epiphytic bacterial taxa from the phylum to genus levels based on an LDA score > 3.5 (*P* < 0.05) (Fig. 4). Key bacterial species with significant abundance differences in the six plants included: *C. demersum* (*Proteobacteria*, *Gammaproteobacteria*, *Pantoea*, *Pseudomonas*, and *Pneumatobacteria*), *H. verticillata* (Comamonadaceae, *Acidophilus*, *Rhizobacteria*, and *Soilobacterium* spp.), *M. verticillatum* (*Proteobacteria*), *P. lucens* (*Microbacterium*, *Branchiobacterium*, *Cyanobacteria* phylum *Roseofilum*, *Polycoccus*, *Proteobacteria*, *Sphingomonas* spp.), *S. pectinata* (*Flavobacterium* spp., *Chloroflexi*, and *Verrucomicrobia*), and *N. marina* (*Pseudomonas* and *Massilia_*sp.). Multiple group comparisons revealed species with significant abundance differences among the six submerged macrophytes (Fig. S3).

Within each group, there were unique bacterial taxa at the species level (Fig. S4). Species present at < 1% were combined as “other.” For each plant species, the bacterial species with the richest proportion was as follows: *C. demersum* (*Spirochaetales_bacterium_NM-380-WT-3C1* [9.88%]), *H. verticillata* (*Variovorax_*sp*._HH01* [55.04%]), *M. verticillatum* (*Actinomyces_*sp.*_S4-C9* [6.53%]), *P. lucens* (*Acinetobacter_*sp.*_ANC_4641* [13.52%]), *S. pectinata* (*Lysinibacillus_telephonicus* [4.18%]), and *N. marina* (*Rhizobium_*sp.*_Leaf68* [13.27%]).

### Analysis of the Main Metabolic Functions of the Epiphytic Bacteria

To elucidate the potential role of epiphytic bacteria in energy metabolism, we compared macro-genomic data with the KEGG database to obtain gene abundances associated with the carbon, nitrogen, and phosphorus cycles. The outcomes are then classified into different pathways.

**Fig. 5:**
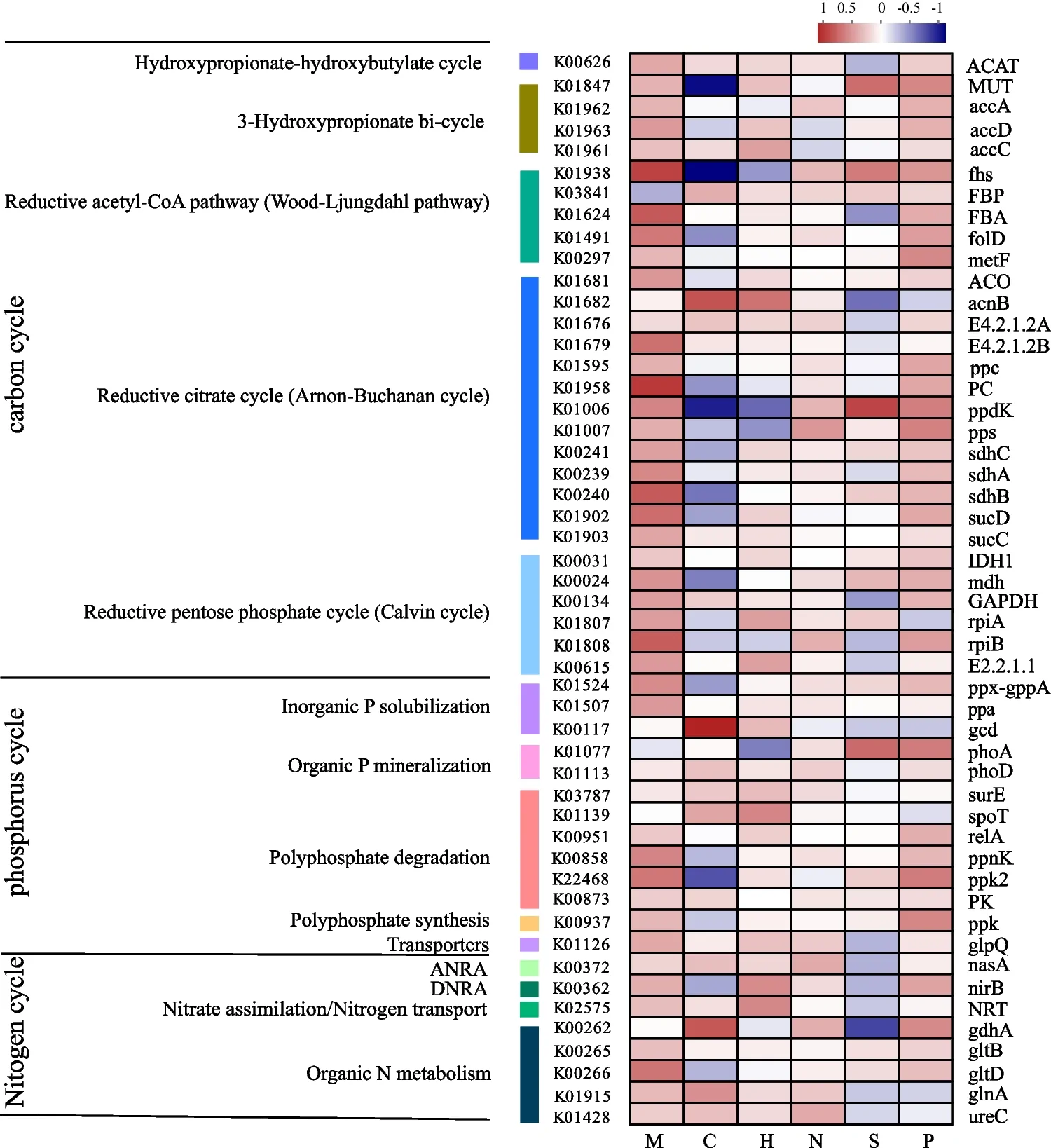
Top 50 functional genes involved in C, N, and P abundance in six plant epiphytic bacteria, with analysis of variance of count data (given using log2 normalized counts) used to compare gene abundance across plants. The depth of color in the heat map represents the abundance of genes. N, *N. marina*; P, *P. lucens*; M, *M. verticillatum*; C, *C. demersum*; H, *H. verticillata*; S, *S. pectinata*

Five carbon fixation pathways were identified, with the reductive citric acid cycle (Arnon-Buchanan cycle), represented by *ACO*, *acnB*, *E4.2.1.2A/B*, *ppc*, *PC*, *ppdK*, *pps*, *sdhA/B/C*, and *sucC/D*, being the most widely distributed. This was followed by the reductive pentose phosphate cycle (Calvin cycle) (*IDH1*, *GAPDH*, *mdh*, *rpiA*, *rpiB*, and *E2.2.1.1*), reductive acetyl-CoA pathway (Wood-Ljungdahl pathway) (*fhs*, *FBP*, *FBA*, *fold*, and *metF*), 3-hydroxypropionate bicycle (*MUT* and *accA/C*), and hydroxypropionate-hydroxybutylate cycle (*ACAT*) (Fig. 5). Overall, the abundance of carbon cycle–encoding genes in the epiphytic bacterial community of *M. verticillatum* exceeded that in the epiphytic bacterial communities of other plant species, especially the gene encoding methylmalonyl-CoA mutase [30]. The gene encoding pyruvate, orthophosphate dikinase (*ppdK*) was significantly enriched in the epiphytic bacterial community of *S. pectinata*, while that encoding aconitate hydratase 2/2-methylisocitrate dehydratase (*acnB*) was enriched in *C. demersum*. Genes involved in these pathways were primarily contributed by *Pseudomonadaceae*, *Micrococcaceae*, *Microbacteriaceae*, and *Sphingomonadaceae* (Fig. S5a).

Four nitrogen metabolic pathways were identified: organic nitrogen metabolism, nitrate assimilation/nitrogen transport, assimilatory nitrate reduction (ANRA), and dissimilatory nitrate reduction (DNRA) (Fig. 5). Enrichment in the gene encoding glutamate synthase (*gltB*) in the epiphytic bacteria of *S. pectinata* suggests a crucial role for the organic nitrogen metabolism pathway in this plant. Nitrogen metabolism in *C. demersum* was influenced by the positive enrichment of genes encoding glutamine synthetase (*glnA*) and glutamate dehydrogenase (NADP^+^) (*gdhA*) (*P* < 0.05). Enrichment of the gene encoding glutamate synthase (NADPH) (*gltD*) in *M. verticillatum* indicates an active role of organic nitrogen metabolism in this plant. *Pseudomonadaceae*, *Microbacteriaceae*, and *Micrococcaceae* were found to be responsible for the organic nitrogen metabolism pathway, while *Pseudomonadaceae*, *Oxalobacteraceae*, and *Bradyrhizobiaceae* were associated with the nitrate assimilation/nitrogen transport (Fig. S5b). Genes encoded by *Microbacteriaceae* and *Micrococcaceae* were linked to the ANRA pathway, and genes encoded by *Micrococcaceae* were associated with the DNRA pathway.

Five phosphorus metabolic pathways were identified: polyphosphate degradation, inorganic P solubilization, organic P mineralization, polyphosphate synthesis, and transporters (Fig. 5). The significant enrichment in the gene encoding quinoprotein glucose dehydrogenase (*gcd*) in *C. demersum* suggests the occurrence of polyphosphate ATP pathway degradation in this plant. Genes encoding NAD^+^ kinase (*ppnK*) and polyphosphate kinase (*ppk2*), responsible for polyphosphate degradation and organic P mineralization, were enriched in *M. verticillatum*, indicating phosphorylation in this plant. The gene encoding alkaline phosphatase (*phoA*) was enriched in *S. pectinate* and that of polyphosphate synthesis (*ppk*) in *P. lucens.* The gene encoding glycerophosphodiester phosphodiesterase (*glpQ*), responsible for transporters, was enriched in *M. verticillatum*. *Pseudomonadaceae* and *Micrococcaceae* were attached to transporters and polyphosphate synthesis pathways, whereas alkaline phosphatases (*phoA/D*) in the organic P mineralization pathway were associated with *Oxalobacteraceae*, *Moraxellaceae*, and *Xanthomonadaceae*. Genes attached to the polyphosphate degradation pathway (*ppnk/ppk2*) were mainly contributed by *Pseudomonadaceae*, *Sphingomonadaceae*, and *Xanthomonadaceae*, while genes involved in the inorganic P solubilization pathway were mainly contributed by *Pseudomonadaceae*, *Xanthomonadaceae*, *Moraxellaceae*, and *Micrococcaceae*. The presence of genes encoded by bacteria showed extensive phosphorus-scavenging mechanisms of these heterotrophic bacteria (Fig. S5c).

## Discussion

In this study, *Proteobacteria* emerged as the most dominant phylum within the epiphytic bacterial communities of the six submerged macrophytes. Particularly noteworthy was the significantly higher abundance of *Proteobacteria* associated with *C. demersum* compared with that in the other five plant species. The α-diversity of the epiphytic bacterial communities exhibited significant differences among certain plant species, with *Proteobacteria* prevailing as the dominant taxon at the phylum level. *Proteobacteria*, known for its wide environmental distribution, plays a crucial role in facilitating bacterial colonization and biofilm formation [12]. It is a common phylum in the plant epiphytic bacteria community [17, 31, 32], as reported in previous studies. Notably, the genus *Pseudomonas* dominated the epiphytic bacterial communities in various plants, such as *C. demersum*, *M. verticillatum*, *P. lucens*, and *S. pectinata*, all exhibiting a robust capability for phosphorus solubilization in eutrophic water bodies [6, 33, 34].

Plant species are an important factor contributing to differences in the community structure of epiphytic bacteria [35]. As an important vegetative organ of submerged macroplants, leaves have a variety of functional characteristics, and the size, shape, and age of leaves also affect the structure and number of attached organisms [36]. Plant complexity has a positive effect on the biomass of surrounding plants [37, 38]. For epiphytic bacterial communities, Levi et al. compared two types of epiphytic plants and their epiphytic bacterial communities, *Callitriche* genus with complex morphology had higher abundance and evenness than *Sparganium emersum* epiphytic bacterial community with simple morphology [14]. Grossart et al. found that highly complex plants would reduce the shear stress of water, thus affecting the richness and diversity of microbial communities [39]. In our study, however, plant morphological complexity was only significantly associated with the diversity of the epiphytic bacterial community.

Furthermore, large freshwater plants can provide nutrients through secretions or the diffusion of photosynthetic products such as polyphenols and cyclic sulfur compounds to produce allelopathy supporting diverse bacterial communities [40, 41] and increasing the diversity of bacterial communities. For example, the secretions of *Hydrilla verticillata* promote positive interactions between bacteria and fungi, increasing the α-diversity of epiphytic bacterial communities [42]. By comparing the biomass differences between natural and artificial substrate communities, authors considered that nutrients and allelopathic substances released by natural plants are the main reasons for the significant differences [37, 43]. Exudate metabolites and secondary metabolites on plant surfaces are not the same [44]. Zhu et al. compared the allelopathic substances of five submerged plants, such as *Sophora* and *Ceratina*, which act as inhibitors or promoters of bacterial growth and lead to the colonization selection of specific epiphytic bacteria [45]. Phenolic compounds in allelopathic substances represent many secondary plant metabolites with antimicrobial activity. Phenolic compounds inhibit the colonization of *Gammaproteobacteria* [46]. Therefore, the epiphytic bacterial communities of submerged plants with different phenolic substrates are significantly different [16].

Submerged epiphytic bacterial communities play a crucial role in nutrient cycling in freshwater lakes. The results of our study indicate that bacterial genes associated with metabolic functions exhibit host plant specificity. Furthermore, the abundance of carbon cycling genes in *M. verticillatum* and *P. lucens* surpasses that observed in other epiphytic bacterial communities. It was concluded that the abundance of plant nutrient cycling–related genes correlates with specific leaf traits, noting that flat-leafed plants demonstrate superior photosynthesis compared to needle-leaf plants [47]. These findings align with Yu et al. who observed significant differences in the functions of epiphytic bacteria on three submerged macrophytes—*H. verticillata*, *Vallisneria natans*, and *Potamogeton maackianus* [10]. Discrepancies in the abundance of metabolic function genes in epiphytic bacteria may be attributed to their physicochemical properties. For example, during carbon cycle activity, *Methylobacterium* spp. (pink-pigmented facultative methylotrophic bacteria) rely on plant leaves to release compounds such as methane and methanol for growth and reproduction. These compounds contribute not only to the oxidative conversion of methane to carbon dioxide but also to the fixation of carbon dioxide by plants. The content of these nutrients on the leaf surface varies according to the plant species [15].

The abundance of genes involved in nitrate reduction was higher in the epiphytic bacteria of *H. verticillata*, and the abundance of genes related to organic nitrogen metabolism in *M. verticillatum* was higher than in other species. Nitrate assimilation/nitrogen transport, DNRA, ANRA, and denitrification have been standardized as nitrate reduction processes, primarily used for amino acid synthesis and promoting microbial metabolism and growth. The enrichment of genes related to the nitrate reducing pathway in the epiphytic bacteria of *H. verticillata* suggests that nitrogen depletion exceeds foreign nitrogen input or biological nitrogen fixation [48]. Jin et al. found that among five submerged aquatic plants, *H. verticillata* had the highest nitrogen removal capacity [49]. Choudhury et al. and Achouak et al. found lower denitrification rates in plants with allelopathy. Functional traits, such as plant biomass and leaf area, are also major factors affecting nitrogen accumulation and denitrification rates [50, 51]. However, this was not in accordance with our results, in which *H. verticillata* exhibited allelochemical effects and biomass and leaf area did not confer a significant advantage among the six plant species. Nitrogen cycling genes in *H. verticillata* were primarily contributed by members of the *Comamonadaceae*, which are common facultative denitrifying bacteria belonging to *Betaproteobacteria* [52]. We hypothesize that these strains play a crucial role in nitrogen degradation by *H. verticillata* and that diazotrophic communities and nitrozyme activities, such as those of *Comamonadaceae*, are significantly influenced by the host plant species [19]. This may explain the higher abundance of genes related to nitrate reduction in *H. verticillata* epiphytes. Phosphorus is an essential nutrient for plant growth, and inorganic phosphorus solubilization, organic phosphorus mineralization, and polyphosphate degradation are processes that convert phosphorus into an effective form for plant utilization. This allows plants to take up inorganic phosphorus, which microorganisms efficiently utilize and immobilize in the plant [53]. *Massilia* sp. was significantly enriched in the *N. marina* epiphytic bacterial community compared with other epiphytic bacterial communities. The positive contribution of phosphorus soluble genes (ppx-gppA, ppa, gcd, etc.) in *Massilia* sp. may explain the high expression of inorganic phosphorus process–related genes [54]. Genes attached to the polyphosphate degradation pathway, such as *ppnk* in *M. verticillatum* and *spoT* in *C. demersum* and *H. verticillata*, were enriched, suggesting that these plants play a substantial role in polyphosphate degradation, leading to the absorption of large amounts of inorganic phosphorus [55]. *Pseudomonas*, known for both accumulating and solubilizing phosphate, enriched in *C. demersum*, *H. verticillata*, and *M. verticillatum*, demonstrates a significant ability to degrade phosphate and performs well in polyphosphate degradation processes.

The composition of epiphytic bacterial communities is a crucial factor influencing the abundance of carbon, nitrogen, and phosphorus cycling genes [56], directly impacting ecosystem functioning [57]. Previous studies have highlighted that foliar nutrient sources regulate microbial growth, select and filter epiphytic bacterial communities, and contribute to differences in these communities on leaf surfaces [1]. For example, differences in the available sugar content on leaf surfaces limit bacterial community size [15], and leached metabolites such as polyphenols are selected for specific bacterial colonization [16]. In addition, leaf functional traits affect the abundance of nutrient cycling–related genes [47]. Different leaf shapes result in variations in light availability and other resources [37], providing additional microhabitats and ecological niches for epiphytic bacteria, thereby influencing their abundance and evenness [14].

## Conclusion

Our findings reveal substantial variations in the abundance of epiphytic bacterial communities among six submerged plant species, while diversity and evenness remained statistically insignificant, and there was a significant correlation between plant diversity and plant complexity. *Proteobacteria* emerged as the predominant phylum, shared by the epiphytic bacterial communities on the leaf surfaces of all six submerged macrophytes. Noteworthy distinctions in the composition of epiphytic bacterial communities were observed among the plant species, spanning from the family to species taxonomic levels. Specifically, the epiphytic bacteria associated with submerged macrophytes, particularly those attached to *M. verticillatum*, assume a pivotal role in facilitating the transport and transformation of carbon, nitrogen, and phosphorus within aquatic ecosystems—a role strongly influenced by the host plant. The results of this study provide a deeper understanding of both the composition and functional aspects of epiphytic bacteria residing on submerged plant leaves, shedding light on their integral roles in freshwater ecosystems.

## Supplementary information

Supplementary file 1 (PDF 13.7 kb)

Supplementary file 2 (PDF 117 kb)

Supplementary file 3 (PDF 272 kb)

Supplementary file 4 (PDF 1.23 mb)

Supplementary file 5 (PDF 674 kb)

Supplementary file 6 (PDF 51.7 kb)

## Data Availability

Data will be made available on request.
