# Supplementary material for: Analysis of diversity and function of epiphytic bacterial communities associated with macrophytes using a metagenomic approach
Source: Microb Ecol. 2024 Jan 29;87:37. doi: 10.1007/s00248-024-02346-7 (PMC10824801; doi:10.1007/s00248-024-02346-7)
Supplement: Supplementary file 2 [file 248_2024_2346_MOESM2_ESM.pdf]

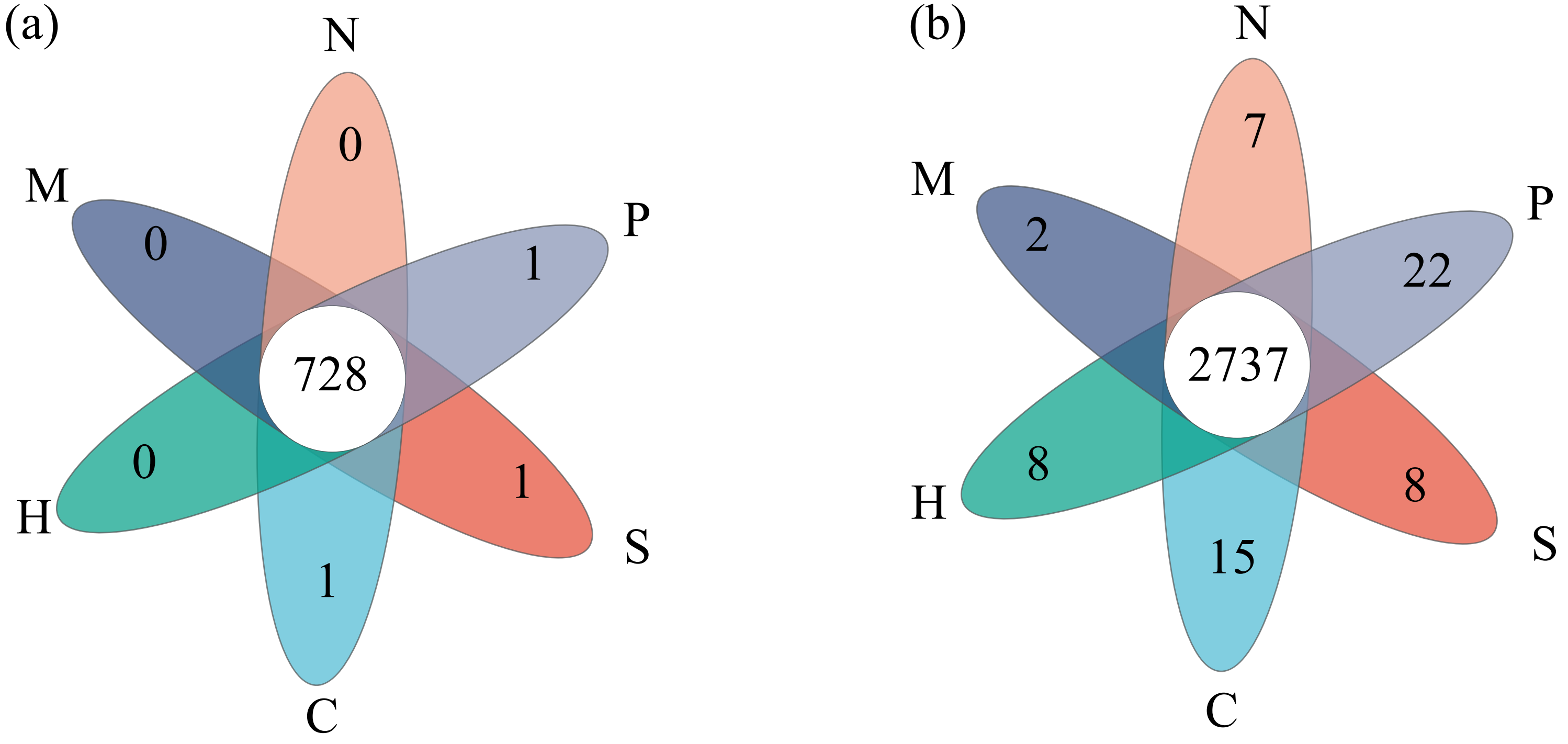

(a)
N
M
P
H
S
C
0
0
1
0
1
1
728
(b)
N
M
P
H
S
C
7
2
22
8
8
15
2737
