# Supplementary material for: Analysis of diversity and function of epiphytic bacterial communities associated with macrophytes using a metagenomic approach
Source: Microb Ecol. 2024 Jan 29;87:37. doi: 10.1007/s00248-024-02346-7 (PMC10824801; doi:10.1007/s00248-024-02346-7)
Supplement: Supplementary file 3 [file 248_2024_2346_MOESM3_ESM.pdf]

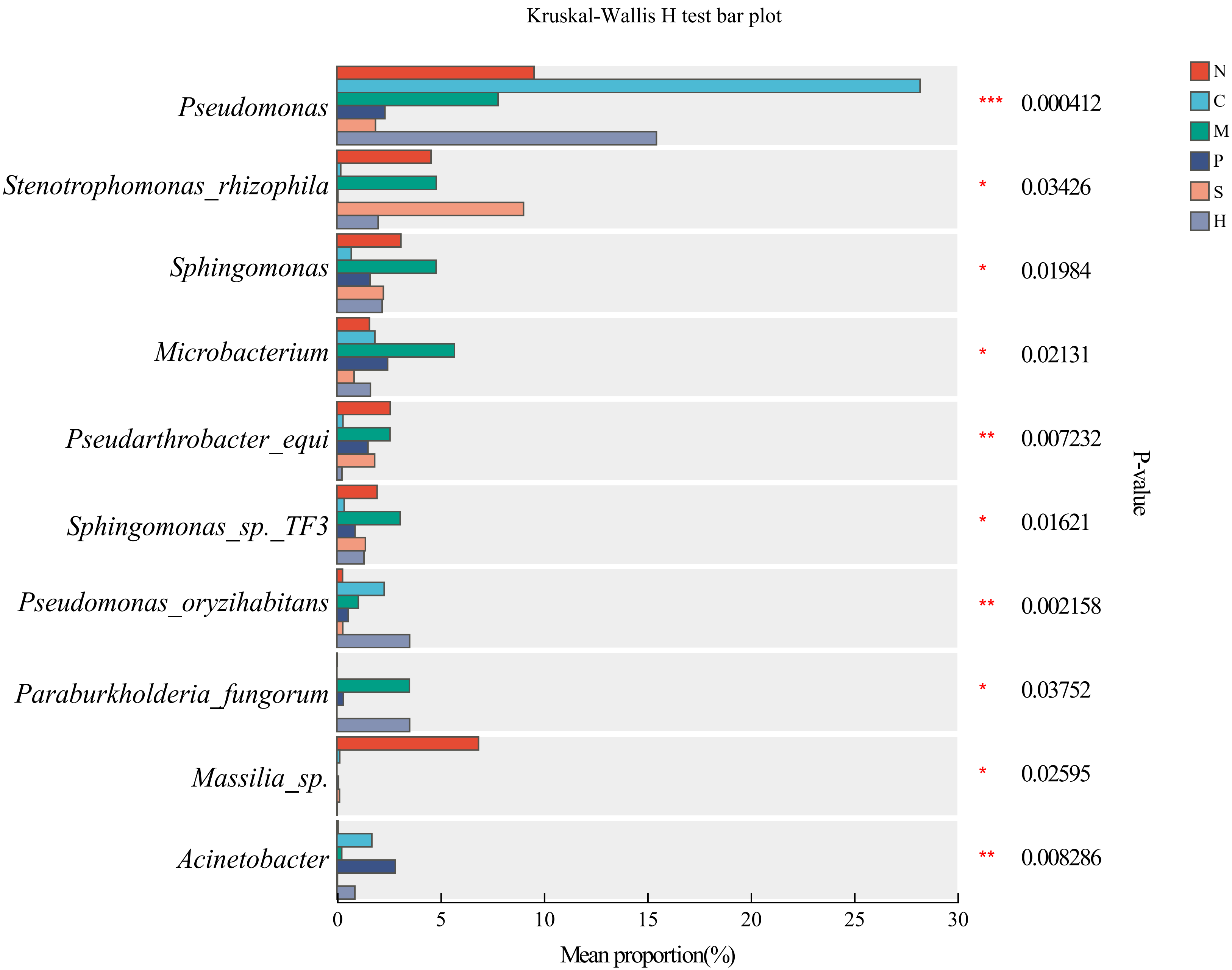

Kruskal-Wallis H test bar plot
Pseudomonas
Stenotrophomonas_rhizophila
Sphingomonas
Microbacterium
Pseudarthrobacter_equi
Sphingomonas_sp._TF3
Pseudomonas_oryzihabitans
Paraburkholderia_fungorum
Massilia_sp.
Acinetobacter
*** 0.000412
* 0.03426
* 0.01984
* 0.02131
** 0.007232
* 0.01621
** 0.002158
* 0.03752
* 0.02595
** 0.008286
P-value
N
C
M
P
S
H
0
5
10
15
20
25
30
Mean proportion(%)
