# Supplementary material for: Analysis of diversity and function of epiphytic bacterial communities associated with macrophytes using a metagenomic approach
Source: Microb Ecol. 2024 Jan 29;87:37. doi: 10.1007/s00248-024-02346-7 (PMC10824801; doi:10.1007/s00248-024-02346-7)
Supplement: Supplementary file 4 [file 248_2024_2346_MOESM4_ESM.pdf]

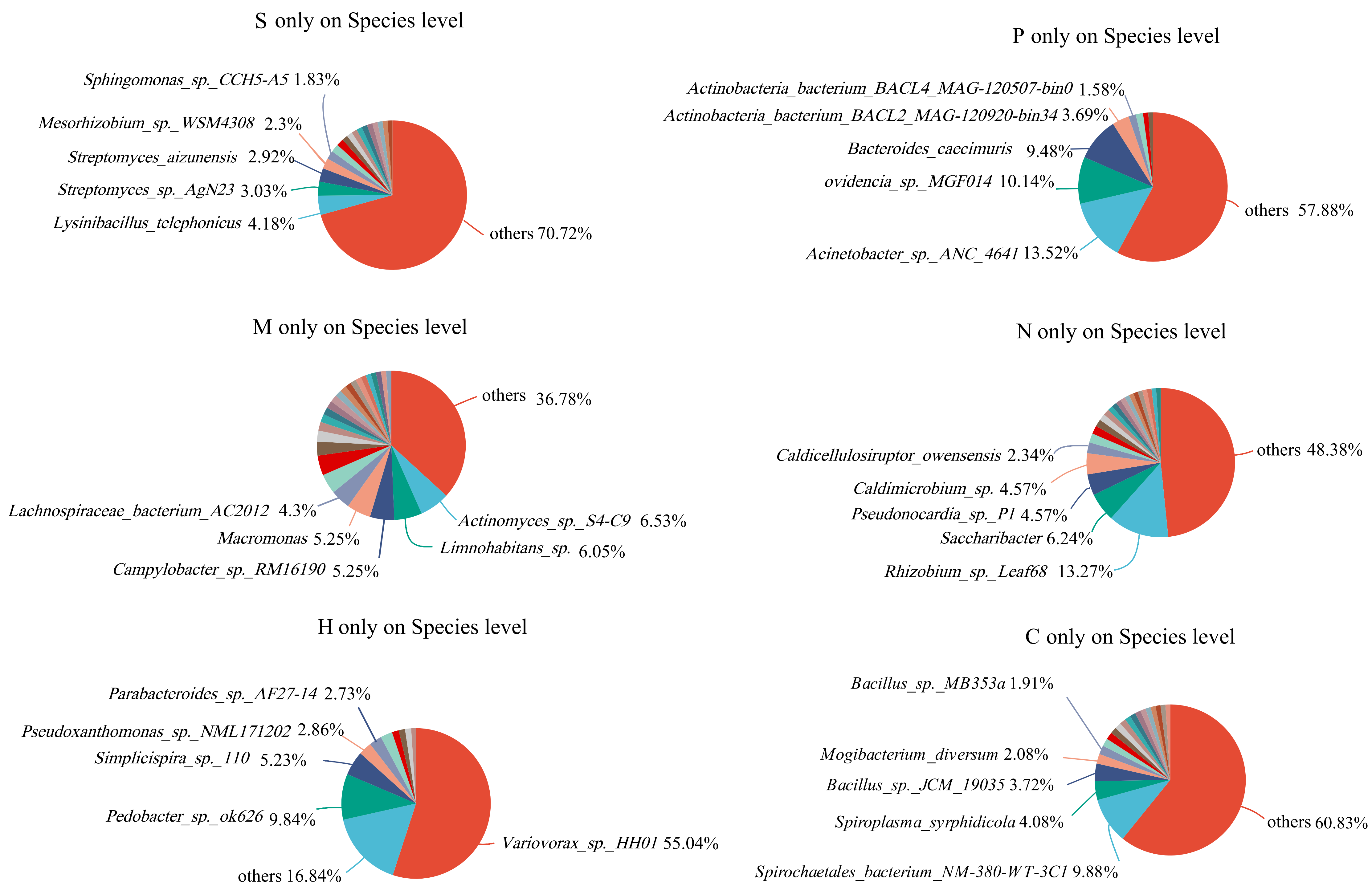
S only on Species level
Sphingomonas_sp._CCH5-A5 1.83%
Mesorhizobium_sp._WSM4308 2.3%
Streptomyces_aizunensis 2.92%
Streptomyces_sp._AgN23 3.03%
Lysinibacillus_telephonicus 4.18%
others 70.72%
P only on Species level
Actinobacteria_bacterium_BACL4_MAG-120507-bin0 1.58%
Actinobacteria_bacterium_BACL2_MAG-120920-bin34 3.69%
Bacteroides_caecimuris 9.48%
ovidencia_sp._MGF014 10.14%
Acinetobacter_sp._ANC_4641 13.52%
others 57.88%
M only on Species level
others 36.78%
Lachnospiraceae_bacterium_AC2012 4.3%
Macromonas 5.25%
Campylobacter_sp._RM16190 5.25%
Actinomyces_sp._S4-C9 6.53%
Limnohabitans_sp. 6.05%
N only on Species level
Caldicellulosiruptor_owensensis 2.34%
Caldimicrobium_sp. 4.57%
Pseudonocardia_sp._P1 4.57%
Saccharibacter 6.24%
Rhizobium_sp._Leaf68 13.27%
others 48.38%
H only on Species level
Parabacteroides_sp._AF27-14 2.73%
Pseudoxanthomonas_sp._NML171202 2.86%
Simplicispira_sp._110 5.23%
Pedobacter_sp._ok626 9.84%
others 16.84%
Variovorax_sp._HH01 55.04%
C only on Species level
Bacillus_sp._MB353a 1.91%
Mogibacterium_diversum 2.08%
Bacillus_sp._JCM_19035 3.72%
Spiroplasma_syrphidicola 4.08%
Spirochaetales_bacterium_NM-380-WT-3C1 9.88%
others 60.83%
