# Supplementary material for: Analysis of diversity and function of epiphytic bacterial communities associated with macrophytes using a metagenomic approach
Source: Microb Ecol. 2024 Jan 29;87:37. doi: 10.1007/s00248-024-02346-7 (PMC10824801; doi:10.1007/s00248-024-02346-7)
Supplement: Supplementary file 5 [file 248_2024_2346_MOESM5_ESM.pdf]

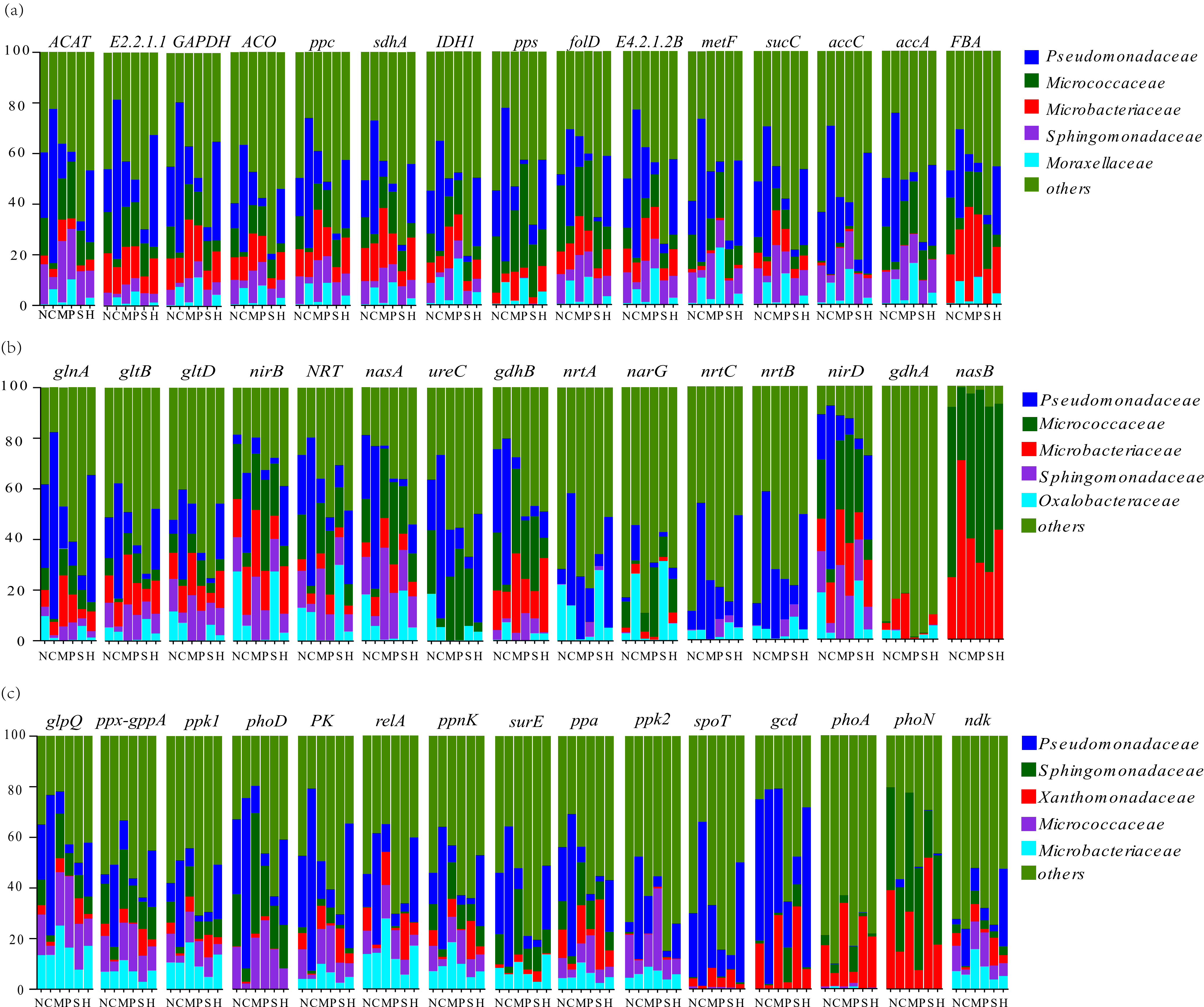
(a)
ACAT
E2.2.1.1
GAPDH
ACO
ppc
sdhA
IDH1
pps
folD
E4.2.1.2B
metF
sucC
accC
accA
FBA
100
80
60
40
20
0
NCMPSH
Pseudomonadaceae
Micrococcaceae
Microbacteriaceae
Sphingomonadaceae
Moraxellaceae
others
(b)
glnA
gltB
gltD
nirB
NRT
nasA
ureC
gdhB
nrtA
narG
nrtC
nrtB
nirD
gdhA
nasB
Pseudomonadaceae
Micrococcaceae
Microbacteriaceae
Sphingomonadaceae
Oxalobacteraceae
others
(c)
glpQ
ppx-gppA
ppk1
phoD
PK
relA
ppnK
surE
ppa
ppk2
spoT
gcd
phoA
phoN
ndk
Pseudomonadaceae
Sphingomonadaceae
Xanthomonadaceae
Micrococcaceae
Microbacteriaceae
others
