# Supplementary figures and images for: Analysis of diversity and function of epiphytic bacterial communities associated with macrophytes using a metagenomic approach
Source: Microb Ecol. 2024 Jan 29;87(1):37. doi: 10.1007/s00248-024-02346-7 (PMC10824801; doi:10.1007/s00248-024-02346-7)

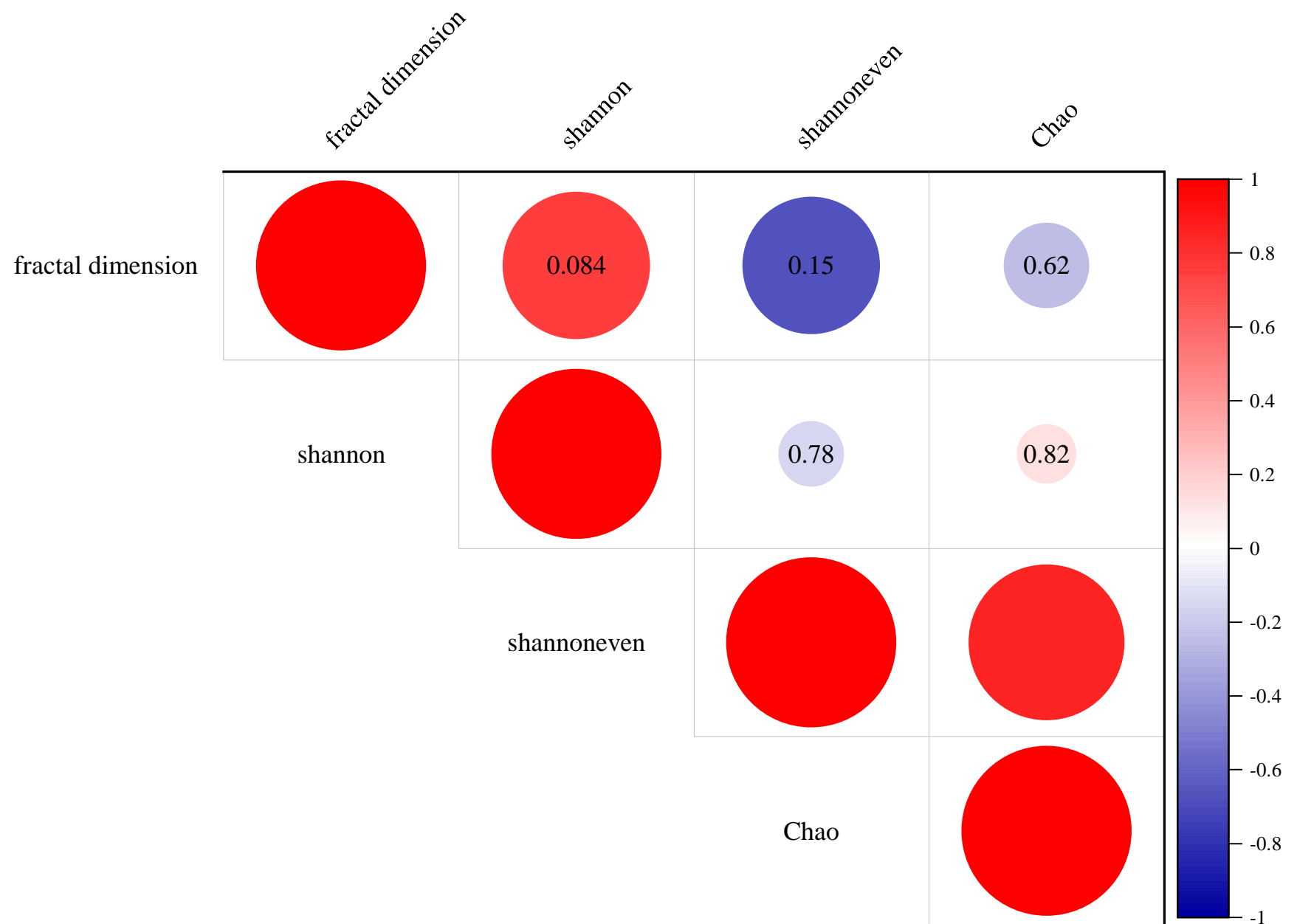

Significant level: 0.05

Supplement: Supplementary file 1 — (PDF 13.7 kb) [file 248_2024_2346_MOESM1_ESM.pdf]

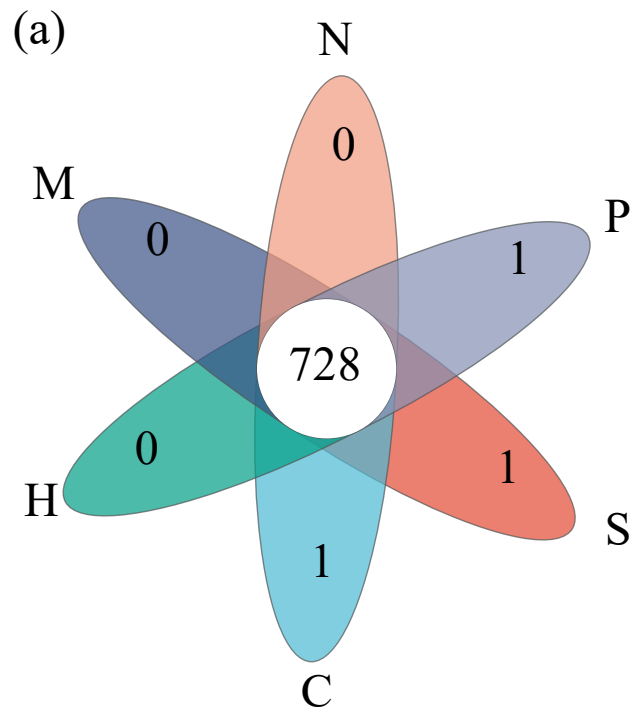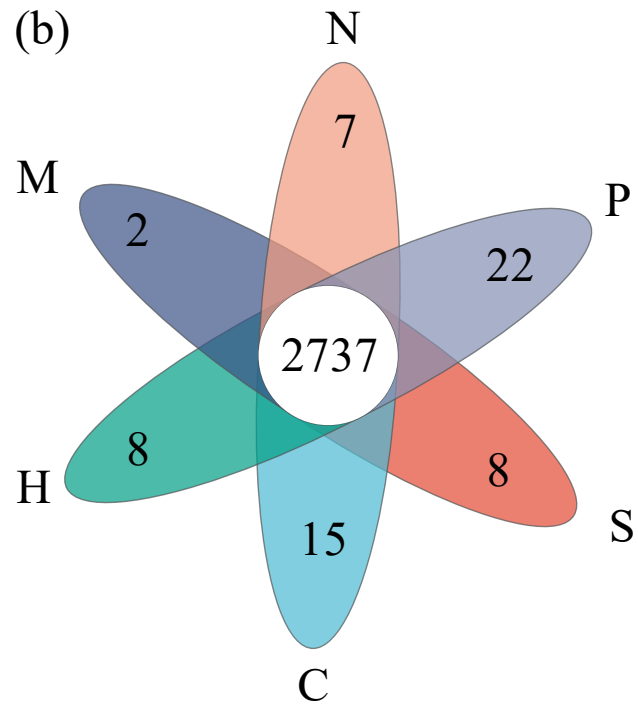

Supplement: Supplementary file 2 — (PDF 117 kb) [file 248_2024_2346_MOESM2_ESM.pdf]

Kruskal-Wallis H test bar plot

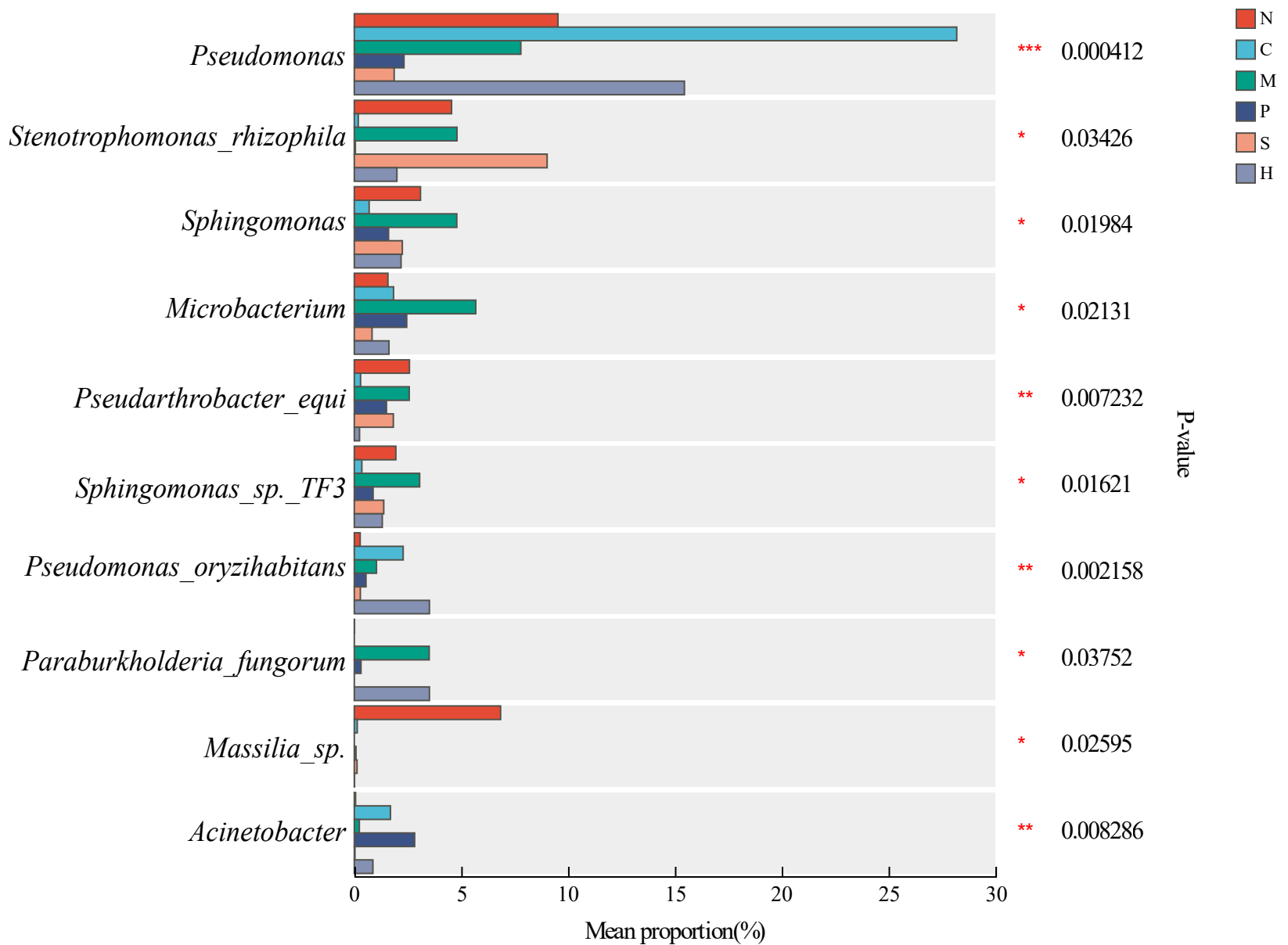

Supplement: Supplementary file 3 — (PDF 272 kb) [file 248_2024_2346_MOESM3_ESM.pdf]
