# Supplementary material for: Analysis of diversity and function of epiphytic bacterial communities associated with macrophytes using a metagenomic approach
Source: Microb Ecol. 2024 Jan 29;87(1):37. doi: 10.1007/s00248-024-02346-7 (PMC10824801; doi:10.1007/s00248-024-02346-7)
Supplement: Supplementary file 6 — (PDF 51.7 kb) [file 248_2024_2346_MOESM6_ESM.pdf]

Table. S1 Leaf morphology description, fractal dimension and water content of different submerged plants

Submerged macrophytes	Leaf profile description	Fractal dimension(D220X)	Moisture content （%）
Myriophyllum verticillatum	Filiform whole fissure, petiolate absent; Lobes 8-13 pairs, alternate, 0.7-1.5 cm long	1.78 ± 0.03	83.9
Ceratophyllum demersum	Lobes filamentous, or filamentous, 1.5-2 cm long and 0.1-0.5 mm wide, apex with white cartilaginous bone, margin with several fine teeth only on one side	1.76 ± 0.04	84.3
Najas marina	Leaf blade linear-lanceolate, slightly curved upward, 1.5-3 cm long, 2 mm or wider, apex with 1 yellowish-brown nematode, margin with 4-10 coarse serrulate on each side, teeth 1-2 mm long	1.56± 0.03	86.9
Stuckenia pectinata	Densely branched, striate, 2~10cm long, 0.5~1mm wide, apex acute, entire margin	1.43±0.03	78.8
*Potamogeton lucen*s	Elliptic alternate leaves (6 ~ 18 cm long, 0.5 ~ 3.5 cm wide), undulating margins, without or with short petioles，	0.88±0.03	79.3
Hydrilla verticillata,	Leaves 4-8 whorled, linear or elongated, 7-17 mm long and 1-1.8 mm wide	1.43± 0.01	78.6
